# Sex Hormones Determine Immune Response

**DOI:** 10.3389/fimmu.2018.01931

**Published:** 2018-08-27

**Authors:** Veena Taneja

**Affiliations:** Department of Immunology and Rheumatology, Mayo Clinic, Rochester, MN, United States

**Keywords:** sex hormones, autoimmune diseases, X-linked genetic disease, immune system, TLRs (toll-like receptors)

Females and males differ in the energy consumption and nutritional requirements which are based on the interactions between environmental factors and sex hormones (1). The studies in early 1940s ascertained that females have enhanced capability of producing antibodies (2, 3). This enhanced immune reactivity in females helps mount an effective resistance to infection and therefore females are less susceptible to viral infections, but can develop immune-pathogenic effects and predisposition to autoimmunity due to hyper immune responses (4, 5). Sex hormones can also control the immune response via circadian rhythm. Many hormones like cortisol, known to regulate T cell mediated inflammation, have a circadian rhythm with a maximum peak at 8:00 a.m. and progressively lower levels as the day progresses (6). Interaction between sex hormones and environmental factors like cigarette smoke and infections lead to variable responses in both genders (5, 7, 8). There is emerging evidence that sex hormones impact microbial composition and the resulting immune response via secondary metabolites binding with receptors like estrogen receptors (ERs), peroxisome proliferator-activated receptors (PPARs) etc. (9). These differences in immune response can lead to variability in disease phenotypes with autoimmunity occurring more often in females and cancers occurring more in males (Figure 1).

**Figure 1:**
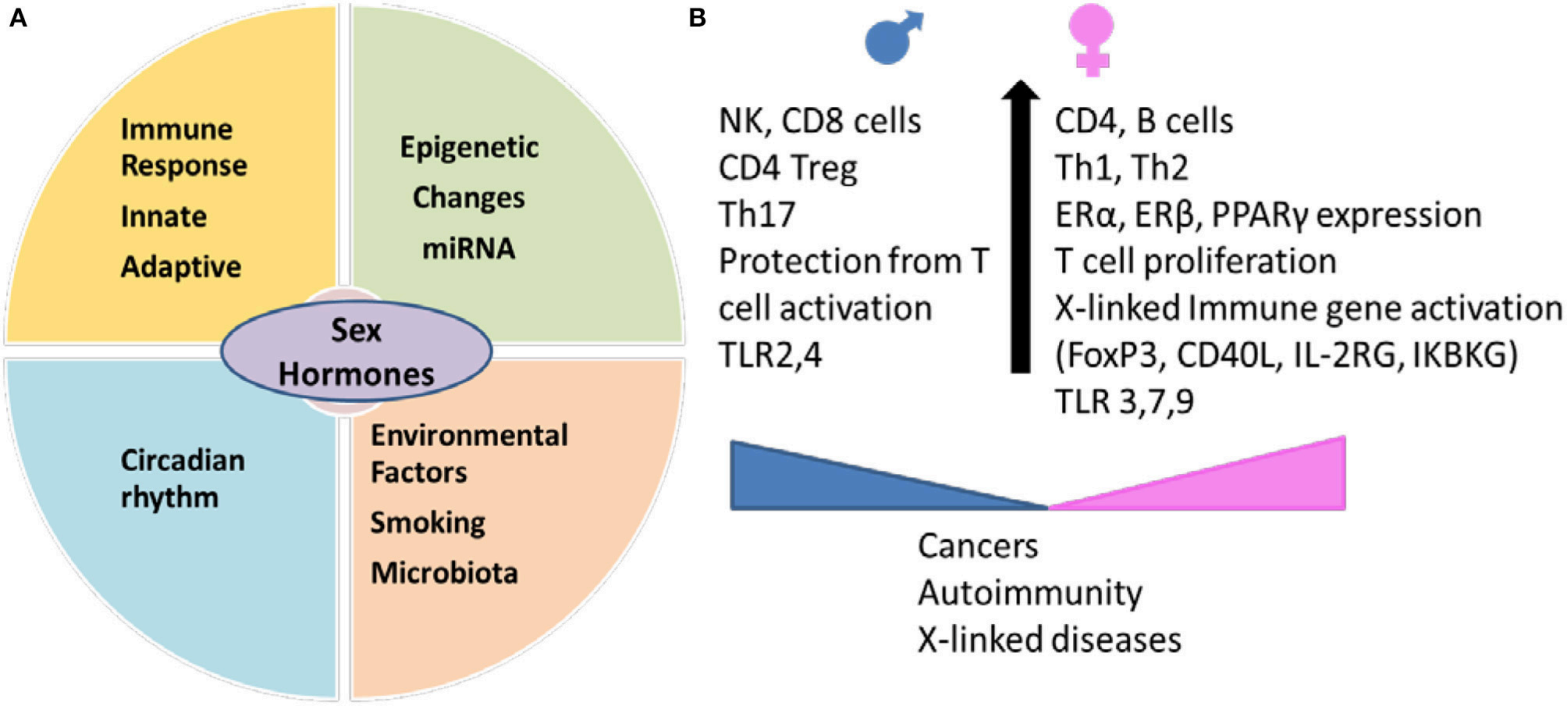
Sex hormones interact with genetic and environmental factors and determine immunity in an individual. **(A)** Environmental factors like smoking and gut microbiome generate sex-hormones dependent immunity leading to differences in circadian rhythm, innate and adaptive immune response and epigenetic changes between males and females. Sexual dimorphism between miRNA expression contributes to sex-specific regulation of function in various tissues. **(B)** Sex determines expression of cell markers involved in innate and adaptive immunity. Females have higher expression of genes on X chromosome which include immune markers like regulatory marker FoxP3, CD40L. Females produce higher Th2 response and antibodies and better protection from infections but the hyperimmune response makes them susceptible to autoimmune diseases. Males generate more of Th17 response and are less likely to develop autoimmunity but have higher percent of non-reproductive cancers. miRNA, microRNA; TLR, Toll like receptor; ER, estrogen receptor; PPAR, peroxisome proliferator-activated receptor; IKBKG, inhibitor of nuclear factor kappa B kinase; IL2RG, interleukin receptor subunit gamma.

## Sex hormones and immunity

Conserved pathogen-associated molecular patterns (PAMPs) of microbes can bind various pathogen recognition receptors like toll like receptors (TLRs). Since TLR expression differs between sexes, TLR3, 7, and 9 are expressed higher in females and TLR2 and TLR4 in males, it can influence strength of TLR-dependent responses. Macrophages from male mice generate a higher TLR4- and TLR2-dependent Th1 response to clear infections, while estrogen regulates immune response via modulation of endosomal TLRs and TLR8 expression thus hormonal balance determines the overall response in females (10–13). TLR3, 7, and TLR9 recognize viral RNA or DNA while TLR2 and TLR4 are known to bind bacterial cell wall proteins. In humans, mononuclear cells from men produce lower levels of type I IFNs in response to TLR7 ligands and higher IL-10 in response to TLR9 ligands as compared to females (14, 15). The differential immune response may also be associated with the differences in immune cell populations between sexes. CD4 and CD8 cells decline during aging in both sexes, though aged women showed lower NK cells and memory Tregs as compared to aged men (16, 17), which may partially explain the sex-biased immune response and cytokine production. Immune responses to environmental factors like infections and vaccinations are also sex-biased (18). Women maintain a high immune reactivity post-viral infections (2). Since females generate higher antibody responses, vaccinations also result in higher antibody levels in women than men and provide efficient protection (19); however this can lead to worse side effects than men due to enhanced immune reactivity. This augmented immune response can perpetuate and precipitate inflammation in many ways including bystander effect, production of pro-inflammatory cytokines and if antigen shares mimicry with a self-protein, an autoimmune response.

Sexual dimorphism in immunity has been described in both arms of immunity, innate and adaptive (20). Generally testosterone has an immunosuppressive effect while estrogen has an immunoenhancing effect on the immune system. Estrogen has been shown to regulate immune response by impairing negative selection of high affinity auto-reactive B cells, modulating B cell function and leading to Th2 response (21, 22). Estrogen influences physiological functions via ERs which are expressed in brain, gut epithelial cells, lymphoid tissue cells as well as immune cells (23, 24). Estrogen also induces T cell homing by enhancing the expression of CCR5, a homing marker (25). Based on the relative numbers of various immune cells in males and females, overall immune response is sexually dimorphic and determines pathogenicity (Figure 1). On the other hand, immune regulation by androgens such as testosterone impacts the immune system by augmenting Th1 response and activation of CD8 cells while down-regulating natural killer (NK) cells response, tumor necrosis factor-alpha (TNFα) and increasing the production of anti-inflammatory IL-10 (26, 27). This is supported by studies showing that *in vitro* presence of testosterone leads to a higher production of Th1 by peripheral blood cells with a higher Th1:Th2 ratio in men (28, 29). The dichotomy of sex-specific response was shown in a humanized mouse model of inflammation where exogenous supply of estradiol and castration in male mice led to an increase in autoimmunity (30) by augmenting Major Histocompatibility Complex II (MHCII) expression and modulating B cells function (31). B cells are targets for treatment in many diseases including rheumatoid arthritis (RA), lupus and multiple Sclerosis (MS). Depletion of B cells in ongoing arthritis in female mice showed higher efficacy as compared to males (32). Similar observations were reported in patients treated with Rituximab where women achieved remission more frequently than men (33). A predominant role of sex hormones has been suggested as the main cause of sex-biased autoimmune diseases like RA and MS (5). Remission of RA and lupus during pregnancy further support a role of female sex hormones in immune response. Although consideration of patient's sex for treatment is not a practice, sex differences in immune response suggest that sex-based treatments would be optimal.

Recently, evidence has emerged on the critical role played by environmental factors like smoking and the gut microbiota in controlling immune responses locally as well as systemically. Gut microbial composition is influenced by many factors including genetic, diet and sex hormones (34–36). Sex-dependent effects of diet were shown on the gut microbial composition in two fish populations (37). In humans, diet-based effects on the microbiome were much more prominent in men than women (38, 39); suggesting diet can further influence sex-bias immune responses by impacting colonic ecosystem. In a study in 1998, women treated with hormonal contraceptives for 3 weeks showed an increase in *Prevotella* species suggesting a direct role of hormones on the gut microbiota (40). The lower abundance of *Prevotella* and *Bacteroides* in females compared to males further supports sex-dependent differences in microbial composition (41), which impact intestinal and systemic immune responses. Metabolites generated by the gut commensals can bind epithelial cells and other immune cells via ERs and PPARs that are expressed differentially in both sexes (42). There is compelling evidence that sex hormones regulate the hippocampal serotonergic system of the gut-brain axis in a sexually dimorphic manner (43). The gut microbiota can impact systemic levels of testosterone via 17β reduction of androgen (44–46) consequently changing the intestinal metabolic landscape. Evidence for this was demonstrated in an experimental model of diabetes where females were protected from diabetes when microbiota from male mice was transferred, which was dependent on an increase in the testosterone levels (47). There is limited information on the mechanism by which microbiome-derived sex steroids impact host immunity. One can speculate that the interaction of sex hormones with environmental factors as well as epigenetic changes caused by the microbiota determine the immune response by cells of innate and adaptive immune cells and the overall sex-biased difference in immune-mediated cytokine responses.

## Genetic factors in sexual dimorphic immunity

Gene diversity or dosage may be one of the factors that can explain the sex-bias in immune responses and female predominance of autoimmune diseases. Women carry two copies of X chromosome, one of which is randomly transcriptionally inactivated while men have only one X. Many genes on X chromosome are associated with regulation of immune functions; IL-2R γ chain, IL-3R α chain, IL-13 α chain, IL-1R associated kinase 1 (IRAK1) TLR7, GATA1, FOXP3, and CD40L. It is surmised that skewed inactivation, mutations or under certain physiological conditions, approximately 10–15% of these genes may be activated (48, 49). In females, maternal or paternal X chromosome inactivation in different cell types combined with the fact that X chromosomes have genes associated with immune functions, it is reasonable to assume that some of these genes may be involved in sex-biased abnormalities in immune responses. X chromosome involvement in sex-bias immunity is supported by the inherited disorders such as Klinefelter with XXY in males and Turner syndrome with XO in females, both with hormonal and immune abnormalities (50). The X chromosome also contains 10% of the microRNA (miRNA) in the human genome as compared to 2 miRNA on the Y chromosome (51, 52). The X-linked miRNAs have also been shown to contribute to sex differences in immune responses, leading to much higher responses in females.

Sex steroid levels change rapidly for women when they are menopausal while in males the change is gradual. While aging is associated with changes in immune cells in both sexes (53), in women heightened immune response and accumulation of antibodies over a period can cause a low grade inflammation which can predispose to sex-bias in inflammatory diseases. MHC molecules present antigens from pathogens and generate immune response. While testosterone has been suggested to decrease the MHC II expression on DCs, estrogen increases the expression (54). As DCs are important for generation of immune responses and T cell differentiation, it may determine the quantitative as well-specific TH cytokines in a sex-specific manner. Thus, even in the presence of similar MHC II, women pay the price of higher incidence of sex-biased diseases but generate a superior response to infections. Interestingly, sex-specific immune response by MHCII molecules in humanized mice showed that males generated higher response to antigens presented by HLA-DQ alleles while females showed higher immune response to HLA-DR-presented antigens (32, 30). HLA-DR and DQ molecules select T cells with different cytokine producing abilities which may dictate the sexually-dimorphic immune response (4). Differential upregulation of MHC expression and antigen presentation leading to differential cytokines milieu in both sexes will determine the outcome of infections and diseases.

Besides the known inherited genes, there is some evidence that non-inherited maternal antigens (NIMA) that are not encoded by the offspring but passed along from the mother may have a role in sex-biased immune response. However, the role of NIMA in various diseases has not been consistent (55). The strongest association for NIMA was observed in RA patients negative for RA-susceptible HLA alleles (56). Besides NIMA, the presence of allogeneic male fetal cells (Fetal microchimerism) in women may also be involved in generating immune response. Although the data is not consistent in most diseases, studies in MS and systemic sclerosis provide some evidence that it is a possibility (57, 58).

The reason why sex-bias immunity exists may lie in the evolution and preservation of mankind. Evolutionarily, during reproductive years, an enhanced response to infections should help maintain health for reproduction. In aged women, reproductive function is not required, enhanced immune reactivity along with changes in immune cells during aging causes sex-specific differences in immunity. The sex-specific expression of genes may explain why women with a similar genetic background show higher immune reactivity or develop autoimmunity at a higher rate than men. Also, the circadian rhythm of sex-hormone-dependent immune system and microbiome could control metabolic profile of an individual. Microbial-metabolites are involved in various signaling pathways as well as immune pathways like differentiation of T cells via binding to receptors of gut immune cells and epithelium. Similar functions also occur in other tissues. Thus, combined with variable X inactivation in cells and pleiotropic nature of many genes, it is likely that sex-hormones impact immune system and its ability to break tolerance to pathogens, environmental or endogenous. Although there is a plethora of evidence suggesting a sex-bias in innate and adaptive immunity that can impact response to infections, vaccinations and onset of various diseases, there is no consensus on treating diseases based on the sex of a patient. The challenge is to be able to define the role of a single receptor or hormone in humans. Animal models have provided some information though more research is required to define the pathways that determine sex-specific immune response during inflammation.

## Author contributions

The author confirms being the sole contributor of this work and approved it for publication.

### Conflict of interest statement

The author declares that the research was conducted in the absence of any commercial or financial relationships that could be construed as a potential conflict of interest.
